# Dental caries experience in children of a remote Australian Indigenous community following passive and active preventive interventions

**DOI:** 10.1111/cdoe.12486

**Published:** 2019-07-21

**Authors:** Jeroen Kroon, Ratilal Lalloo, Santhosh K. Tadakamadla, Newell W. Johnson

**Affiliations:** ^1^ School of Dentistry and Oral Health Griffith University Gold Coast Queensland Australia; ^2^ School of Dentistry The University of Queensland Brisbane Queensland Australia; ^3^ Menzies Health Institute Queensland Griffith University Gold Coast Queensland Australia; ^4^ Faculty of Dentistry, Oral and Craniofacial Sciences King's College London London UK

**Keywords:** children, community water fluoridation, dental caries, Indigenous people, prevention, remote

## Abstract

**Objectives:**

To report on changes in dental caries experience in children of a remote Indigenous community following 6 years of passive preventive intervention (PPI) and 2 years of active preventive intervention (API).

**Methods:**

Five consecutive cross‐sectional surveys were conducted on 4‐ to 15‐year‐old school going children between 2004 and 2017 following phases of Community Water Fluoridation (CWF), post‐cessation of CWF and API. Following treatment of any cavities present, API included selective placement of fissure sealants (FS) and an annual application of povidone‐iodine (PI) and fluoride varnish (FV). The World Health Organization's (WHO) “Oral Health Surveys – Basic Methods (4th Edition)” methodology was used in the first two and the International Caries Detection and Assessment System (ICDAS‐II) in the latter three surveys. ICDAS‐II codes of 3‐6, representing advanced caries, were combined to allow comparison to the decayed component of the DMF caries index.

**Results:**

Age‐weighted mean dmft decreased by 37.7% in the deciduous (DD) and DMFT by 35% in the permanent (PD) dentitions between the pre‐ and post‐CWF surveys, followed by increases of 25% and 7.7%, respectively, between the 1‐year and 4‐year post‐CWF surveys. After 2 years of API, mean dmft decreased by 14.3% and DMFT by 7.1%. Untreated dental caries however remained a concern in the DD and PD during both phases of PPI and of API. The decline in caries experience for both dentitions following 2 years of API exceeded that for the 6‐year period of PPI.

**Conclusions:**

The annual reductions in caries experience of 7.2% (DD) and 8% (PD) during the phase of API exceeded annual decreases of 4.7% (DD) and 4.6% (PD) during the phase of PPI. Due to remoteness, cost and logistics in ensuring long‐term viability of API programmes, CWF remains necessary in this type of community.

## 1 INTRODUCTION

Globally, between 1990 and 2015 the number of people with untreated oral disease is estimated to have increased from 2.5 to 3.5 billion.^1^ Between 1990 and 2017, oral diseases remained one of the two most prevalent causes of the global burden of disease for all ages and sexes combined, despite decreasing by 5.5% over this time.^2^

In the Australian National Child Oral Health Survey (NCOHS) 2012‐14, 27.1% of 5‐ to 10‐year‐olds and 10.9% of 6‐ to 14‐year‐olds presented with untreated dental caries in their deciduous dentition (DD) and permanent dentition (PD), respectively. This was approximately 50% higher for Indigenous compared to non‐Indigenous children and consistently higher again for remote/very remote communities.^3^ A higher burden of dental caries has also been reported for adult Australian Indigenous people^4^ as well as Indigenous communities globally.^5^, ^6^, ^7^

Active and passive preventive measures are defined in terms of the amount of action required to produce benefit. Whereas passive preventive intervention (PPI) protects individuals without any cooperation or action on their part, active preventive intervention (API) not only requires individual action, but often trained personnel, facilities and resources.^8^ For the purpose of this paper, Community Water Fluoridation (CWF) will be regarded as PPI, whereas measures such as fissure sealants (FS), topical povidone‐iodine (PI) and fluoride varnish (FV) applications are examples of API.

Community Water Fluoridation is widely regarded as a safe and effective evidence‐based intervention for the prevention of dental caries, as confirmed recently by a 2018 American Association for Dental Research (AADR) policy statement.^9^ The Australian National Health and Medical Research Council (NHMRC) Information Paper on Water Fluoridation (2017) stated that CWF reduced tooth decay by between 26% and 44% in children, teenagers and adults.^10^ A systematic review published in 2015 confirmed that CWF decreases tooth decay and increases the number of children free of caries in both dentitions, although the authors concluded that much of the evidence was of low quality and that many studies were conducted before 1975.^11^ A critique of this review warned against concluding that CWF was of dubious benefit as all other authoritative reviews have found it to be effective at reducing dental caries in both dentitions.^12^ A United States study including child and adolescent populations reported in 2018 that greater availability of CWF was associated with significantly lower levels of dental caries in both groups.^13^ A consistent association between lifetime exposure to CWF and caries experience was found in both dentitions of Australian children^14^ and a significant decrease in caries experience was reported for a low socioeconomic community in Queensland only 36 months after the introduction of CWF.^15^ The York Report concluded that cessation of CWF resulted in a narrowing of the difference in caries experience between the fluoridated and nonfluoridated communities over time,^16^ as confirmed by a recent systematic review.^17^

Active preventive intervention such as FS, PI and FV has each been found to be effective in reducing dental caries.^18^, ^19^, ^20^, ^21^, ^22^ In Australia, all of these require trained oral health workers and appropriate facilities. As the wider literature indicates that regular re‐application is desirable, this is difficult to sustain in resource‐constrained remote communities.

Following a 2004 survey of children in a remote Indigenous community consisting of five small towns, all within 20 km of each other, in the Northern Peninsula Area (NPA) of Far North Queensland (FNQ) Australia, dental caries experience of 6‐ and 12‐year‐olds was more than twice the Queensland average and more than four times greater than the Australian average.^23^ The Bamaga Hospital has a 2‐chair dental clinic with oral health staff from Thursday Island providing a dental service in this facility for a few days every fortnight. Children in need of emergency care are transported by ferry to Thursday Island.

The aim of this investigation was to report on changes in dental caries experience in children of this community following 6 years of PPI and 2 years of API, spanning 2004‐2017.

## 2 METHODS

Ethics approval was granted by the Griffith University Human Research Ethics Committee (HREC), the FNQ HREC, the Department of Education and Training (Queensland Government) and the Torres and Cape Hospital and Health Service (TCHHS). All surveys were conducted with the full understanding and written consent of parents/guardians of children from the three school campuses in the NPA of FNQ.

Community Water Fluoridation was introduced in 2005 but was ceased in 2011. Enquiries of the company providing and maintaining the reticulated water system in the communities elicited “technical reasons” for ceasing to add fluoride. Funds from the NHMRC Project Grant (APP1081320) enabled us to provide treatment to consenting children at baseline in 2015 and API consisting of selective placement of FS (Conseal F: SDI limited) followed by application of PI (PDI PVP Iodine swab sticks: Professional Disposables International Inc) and FV (Duraphat: Colgate‐Palmolive Pty Ltd) on completion of treatment. An annual re‐application of PI and FV was provided at the 1‐year (2016) and 2‐year (2017) follow‐up visits as per the published protocol.^24^

**Figure 1:**
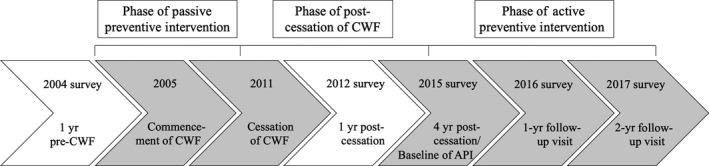
A timeline of surveys and the phases of PPI, post‐cessation of CWF and API between 2004 and 2017

Figure 1 presents a timeline of the five consecutive cross‐sectional surveys and the phases of PPI, post‐cessation of CWF and API between 2004 and 2017. The 2004 survey was conducted by a single calibrated examiner in a dental van with the aid of a dental chair and light.^23^ The 2012 and 2015‐2017 surveys were conducted by a team of two to four calibrated examiners in a specially set‐up classroom using mobile, reclinable dental chairs with fixed‐ and head‐LED lights.^24^, ^25^ The World Health Organization's (WHO) “Oral Health Surveys – Basic Methods (4th Edition)” methodology was used in the 2004 and 2012 surveys.^26^ The International Caries Detection and Assessment System (ICDAS‐II) was used in the 2015‐2017 surveys.^27^ ICDAS‐II codes 3‐6, representing advanced caries, were combined to allow comparison to the decayed component of the DMF caries index.^28^, ^29^, ^30^

Caries experience was calculated for 4‐ to 12‐year‐olds (DD) and 5‐ to 15‐year‐olds (PD) as mean decayed, missing and filled teeth (dmft/DMFT) scores and percentage of children with caries experience. The Significant Caries Index (SiC) is an indication of the high caries risk group in a population and is calculated from dmft/DMFT scores.^31^ SiC_10_ and SiC_30_, respectively, represent 10% and 30% of the population with the highest dmft/DMFT scores.

## 3 RESULTS

Combined demographic information for the five small towns in the NPA of FNQ, obtained from 2006, 2011 and 2016 census data, show that the population gradually increased from 1940 in 2006 to 2799 in 2016 with a median weekly household income of AU$1,027.49. Means of 48.5% male and 51.5% female, 87.5% Indigenous, a median age of 22 and 22.9% between the ages of 5‐14 were found over this time. The number of 5‐ to 14‐year‐olds varied from 493 in 2006 (25.5% of the total population) to 487 in 2011 (21.3%) and 626 in 2016 (22.3%).^32^ These indicators over three censuses are indicative of a stable community, which then allows for comparison between consecutive cross‐sectional surveys.

The number of 4‐ to 15‐year‐olds consenting to the first three surveys was 486 (2004), 324 (2012) and 401 (2015). Based on school records, this represents respective participation rates of 82% (2004), 60% (2012) and 68% (2015). Two consent processes were required for the phase of API: (a) for epidemiological examination and (b) for treatment of existing dental caries, selective FS, PI and FV application. Whereas the majority of parents/guardians consented to their children participating in the survey, consent for treatment and the API was much lower. Children who were not consented to treatment formed a natural comparison group.^24^ Only children who consented to the treatment and API (253 in 2016; 200 in 2017) are included in the results presented here.

**Figure 2:**
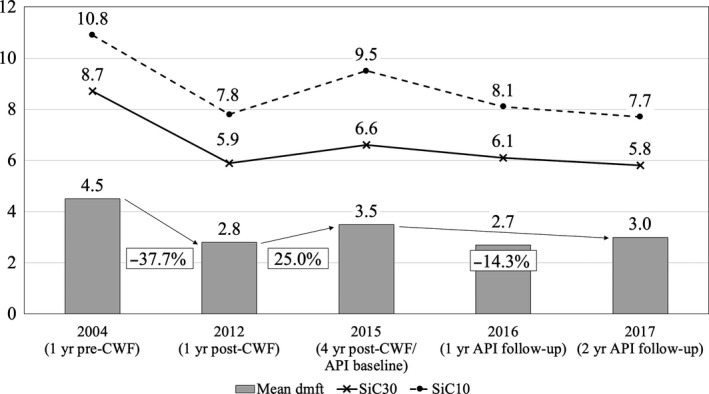
Mean dmft and SiC for the phases of PPI, post‐cessation of CWF and API for 4‐ to 12‐y‐olds (DD)

**Figure 3:**
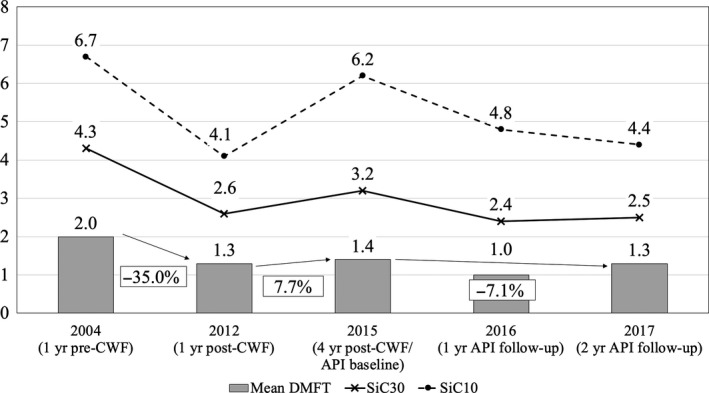
Mean DMFT and SiC for the phases of PPI, post‐cessation of CWF and API for 5‐ to 15‐y‐olds (PD)

Table 1, Figures 2 and 3 present caries experience (mean dmft/DMFT; percentage of children with caries experience) and SiC scores for the DD and PD for the phases of PPI, post‐cessation of CWF and API.

**Table 1:** Caries experience for the phases of PPI, post‐cessation of CWF and API

	Deciduous dentition (4‐ to 12‐y‐olds)						Permanent dentition (5‐ to 15‐y‐olds)					
	n	dt (SD)	mt (SD)	ft (SD)	dmft (SD)	dmft > 0 (%)	n	DT (SD)	MT (SD)	FT (SD)	DMFT (SD)	DMFT > 0 (%)
2004: 1 y pre‐CWF	393	3.5 (4.0)	0.4 (1.4)	0.6 (1.4)	4.5 (4.6)	65.6	468	1.3 (2.2)	0.1 (0.5)	0.6 (1.4)	2.0 (2.9)	54.9
2012: 1 y post‐CWF	263	2.5 (3.0)	0.2 (1.0)	0.1 (0.4)	2.8 (3.2)	63.8	309	1.1 (1.9)	0.1 (0.4)	0.1 (0.4)	1.3 (2.2)	44.8
Difference between 2012 and 2004 (Phase of PPI)					−1.7 −37.7%	−1.8					−0.7 −35.0%	−10.1
2015: 4 y post‐CWF/Baseline of API	284	2.9 (2.9)	0.2 (0.8)	0.4 (0.9)	3.5 (3.3)	75.7	333	1.2 (2.0)	0.0 (0.3)	0.2 (0.7)	1.4 (2.4)	48.0
Difference between 2015 and 2012 (Phase of post‐cessation of CWF)					0.7 25.0%	11.9					0.1 7.7%	3.2
2016: 1‐y API follow‐up	87	1.3 (2.2)	0.2 (0.9)	1.2 (1.6)	2.7 (3.0)	65.5	120	0.4 (0.7)	0.1 (0.5)	0.5 (1.5)	1.0 (2.1)	34.2
2017: 2‐y API follow‐up	63	1.6 (2.0)	0.3 (0.7)	1.1 (1.4)	3.0 (2.9)	73.0	114	0.7 (1.0)	0.1 (0.4)	0.5 (1.0)	1.3 (1.8)	46.5
Difference between 2017 and 2015 (Phase of API)					−0.5 −14.3%	−2.7					−0.1 −7.1%	−1.6

Mean dmft varied from 4.4 (2004) to 2.7 (2016) and mean DMFT from 2.1 (2004) to 1.0 (2016). Untreated decayed teeth (dt/DT) comprised the major proportion of the mean dmft/DMFT scores in all surveys. The percentage of children with caries experience ranged from 63.8% (2012) to 75.7% (2015) for the DD and from 34.2% (2016) to 54.9% (2004) for the PD. For both dentitions, SiC_10_ and SiC_30_ decreased between 2004 and 2017. In 2017, SiC_10_ and SiC_30_ were 2.6 and 2 times higher, respectively, than the mean dmft and 3.7 and 2.1 times higher than the mean DMFT.

For both the DD and PD, a significant decrease in caries experience was observed following 6 years of CWF (PPI).^25^ This was followed by a rebound in the phase of post‐cessation of CWF, more so in the DD than the PD, and a decrease in caries experience over the 2 years of API.

Annual changes in caries experience were calculated as the percentage change in caries over the full period of the intervention, divided by the time in years the intervention was applied. During the phase of API annual reductions in caries experience of 7.2% (DD) and 8% (PD) were found, exceeding annual decreases of 4.7% (DD) and 4.6% (PD) during the phase of PPI.

## 4 DISCUSSION

Aboriginal and Torres Strait Islander (Indigenous) peoples are recognized as priority populations in both Australia's National Oral Health Plans.^33^, ^34^ The Australian NCOHS 2012‐14 reported that nearly 60% of 5‐ to 8‐year‐old Indigenous children experienced dental caries in the DD. This was lower for 9‐ to 14‐year‐old Indigenous children in the PD (46%).^35^ More Indigenous children (44% for the DD; 22.9% for the PD) presented with untreated decayed teeth compared to non‐Indigenous children (25.9% for the DD; 10.1% for the PD).^3^ The Queensland Child Oral Health Survey (QCOHS) 2010‐2012 reported similar findings with 48% of Indigenous 5‐ to 10‐year‐olds (DD) and 20.4% of 6‐ to 14‐year‐olds (PD) presenting with untreated decayed teeth, compared to 28.4% (DD) and 11.7% (PD) for non‐Indigenous children.^36^ A 2018 report on the health of Queenslanders found dental decay to be higher than the state average in the TCHHS where our study community is located.^37^ In our study, the percentage of children with caries experience was similar to the QCOHS results in the DD, whereas lower mean dmft scores, and higher percentage of children with caries experience/mean DMFT scores were found in the PD. In line with other Australian studies in Indigenous populations, our findings confirm that dental caries, especially untreated decay, remains a significant burden of disease in our community as well.

Australian evidence suggests that longer lifetime exposure to CWF resulted in substantially lower caries experience in younger rural adults.^38^ Further analysis of the Australian NCOHS 2012‐14 found consistent associations between lifetime exposure to CWF and childhood caries.^14^ The QCOHS 2010‐2012 reported findings by four geographical regions with lower caries experience reported for Townsville (fluoridated since 1964) compared to Brisbane/South‐East Queensland (fluoridated since 2009) and the remainder of Northern Queensland (nonfluoridated).^39^ Data from our community show a decrease in mean dmft/DMFT, percentage of children with caries experience and SiC for both dentitions for the phase of PPI when CWF was in place (2005‐2011).^25^

Evidence shows that over time cessation of CWF results in a narrowing of the difference in caries experience between fluoridated and nonfluoridated communities.^16^ A recent systematic review concluded that an increase in dental caries occurs post‐CWF cessation, but that the effect is not uniform or inevitable.^17^ In our study we noted an increase in mean dmft/DMFT scores, percentage of children with caries experience and SiC for the phase of post‐cessation of CWF (2012‐2015), but less so in the PD compared to the DD. As the results presented were pooled for all ages with DD (4‐12 years) and with PD (5‐15 years), this difference can be explained by the PD of older children having been exposed to CWF for longer compared to the DD in younger children.

Fissure sealants, PI and FV have all been reported to have significant impact in preventing dental caries. A systematic review reported that FS reduced dental caries on permanent occlusal surfaces between 11% to 51% in children and adolescents 2 years after application^40^ and that reductions in caries experience of between 37% (DD) and 43% (PD) could be achieved by regular application of FV in studies of a duration of between 1‐5 years.^41^ It is recommended that FV and PI should be applied 2‐3 times a year to be most effective^22^, ^40^ with a combination of FV and PI reported to be more effective than FV alone.^20^ In our study, the phase of API (2015‐2017) consisted of treatment of dental decay including selective FS at baseline, followed by application of PI and FV on completion of treatment, with re‐application of PI and FV at the 1‐ and 2‐year follow‐up visits.^24^ API led to a decrease in mean dmft/DMFT, percentage of children with caries experience and SiC in both dentitions. However, the viability and sustainability of API in remote communities, such as that studied here, remain unanswered. A possible option would be to train other health workers, even members from the community, to apply FV and PI more frequently, if permitted by relevant legislation.

Untreated dental caries remained a concern in both dentitions during both phases of PPI and API. Whilst annual API was effective, due to remoteness, cost and logistics in ensuring long‐term viability of such programmes, CWF remains necessary in this type of community. Since substantial dental health disparities and inequalities in access to dental care currently exist in more regional and remote communities, such as the one we studied, there is justification for extending coverage to include all Australians, even when cost‐effectiveness seems less favourable in more remote and smaller communities.^42^ The savings in treatment are greater than the cost of CWF for communities with more than 1000 residents, with the benefit increasing for larger communities.^43^, ^44^ Both an Australian and New Zealand study concluded that extending coverage of CWF to communities of at least 1000 people will result in cost savings to the health sector.^42^, ^45^ Under current Queensland government legislation, the decision to implement CWF rests with local authorities^46^ with many opting out since the legislation changed. Following on recent research developments and persistent challenges to CWF, the AADR published a policy statement which supports its safety and efficacy.^9^ It similarly continues to be endorsed and supported by the Australian NHMRC.^10^ CWF is also considered a cost‐effective method of caries prevention.^42^ It is therefore important that local leaders, community health organizations and the community should lobby for the re‐implementation of CWF in this community.

The time series analysis of this community assumes that the before‐after changes were due to PPI and API and would not have occurred without their introduction. Comparing caries experience as part of this analysis does not take other caries associated factors into consideration. Although aspects of diet and oral hygiene behaviours were not reported in the 2004 and 2012 surveys, during the phase of API, data on diet were collected for variables that could have a confounding effect. Basic groceries and perishable foodstuffs are imported by sea approximately every 2 weeks and are readily available in local stores. Items high in free sugars are abundant in the community. Whilst fresh vegetables and fruit were reasonably priced, it is clear that most children prefer the widely available carbonated drinks and convenience foods. Consumption of sugar‐sweetened beverages is of serious concern in Indigenous communities and in remote settings across Australia, with high consumption increasing especially during adolescence.^47^, ^48^ Since the association between diet and oral (and general) health is well recognized, healthy eating should be emphasized as part of health promotion programmes in this community. Dental services to this community, which could have influenced the results over this time series analysis, however, have not changed considerably, except when treatment was provided to consenting children at baseline of the API in 2015.

Limitations: School absenteeism was common in this community, and this has limited the number of children able to be included, especially across all instances of data acquisition. The 2017 annual report for the three school campuses shows attendance rates around 70% since 2015.^49^ Community efforts did however result in reasonable attendance rates for all the surveys conducted between 2004 and 2017. A further limitation might be the use of the WHO's “Oral Health Surveys – Basic Methods (4th Edition)” methodology in the 2004 and 2012 surveys,^26^ whilst for the 2015‐2017 surveys ICDAS‐II was used,^27^ although ICDAS‐II codes of 3‐6 are accepted as being indicative of advanced caries to enable comparison to the dt/DT component of the DMF caries index.^28^, ^29^, ^30^ No control groups for the 2004, 2012 and 2015 surveys was another limitation. Children who were not consented to treatment during the phase of API formed a natural comparison group.

## 5 CONCLUSIONS

Dental caries remains a significant problem in this remote Indigenous community despite consecutive phases of PPI and API. Continuing efforts to lobby for the re‐implementation of CWF are essential, as are addressing social determinants of health, especially related to diet.

## AUTHOR CONTRIBUTIONS

NWJ leads the project as PI. All authors contributed to conception and design, including participation in field work and data acquisition. JK undertook initial data analysis supported by RL and ST. All authors participated in data interpretation. JK wrote the first draft of the paper which was critically reviewed by all authors, who accept joint responsibility for content.
